# Supplementary material for: Protoplast-Based Regeneration Enables CRISPR/Cas9 Application in Two Temperate Japonica Rice Cultivars
Source: Plants (Basel). 2025 Jul 5;14(13):2059. doi: 10.3390/plants14132059 (PMC12251794; doi:10.3390/plants14132059)
Supplement: Supplementary file 1 [file plants-14-02059-s001.zip › 21. Platino-INIA.pdf]

CEREALES

Arroz grano corto ancho Platino-INIA

NUEVAS VARIEDADES VEGETALES

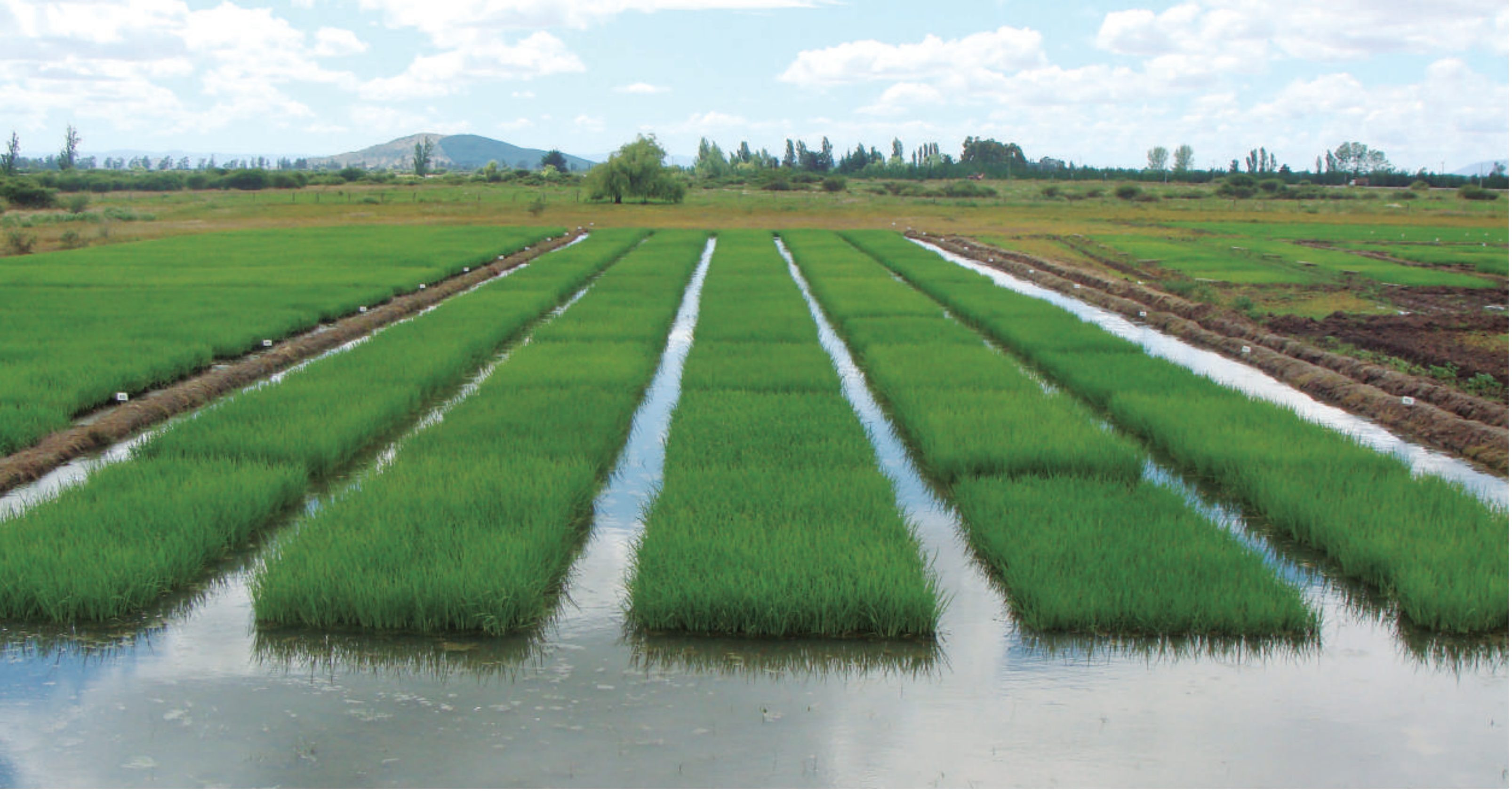

Tecnologías INIA | Unidad de Gestión de la Innovación | Instituto de Investigaciones Agropecuarias (INIA) | 2024

Instituto de Investigaciones Agropecuarias

Arroz grano corto ancho Platino-INIA

DESCRIPCIÓN DEL PRODUCTO

Platino-INIA posee grano medio. Es una variedad semi-tardía, de menor altura y de mayor o similar rendimiento en comparación con la variedad Oro, pero de mejor calidad. Presenta un grano translúcido, con bajo porcentaje y grado de panza blanca; características muy apreciadas en este tipo de grano. La menor altura de planta —respecto de la variedad Oro— confiere a Platino-INIA una mayor tolerancia a la tendedura.

USOS Y APLICACIONES

Por sus características de grano corto y textura más compacta y húmeda en la cocción, Platino-INIA está orientado a la industria de alimentos procesados, específicamente postres (arroz con leche) y para elaboración de sushi, risotto y paella, entre otros.

POTENCIAL DE MERCADO

Se recomienda en toda la zona arrocera, que actualmente se extiende desde la comuna de Linares por el norte hasta la comuna de San Carlos por el sur. El mercado para la variedad Platino-INIA en Chile —si bien es acotado— presenta una oportunidad de comercialización en un segmento de consumidores dispuesto a explorar las bondades culinarias de este tipo de arroz. Las empresas asociadas a la producción y comercialización de arroz en Chile están fomentando el uso de este tipo de granos, para diversificar el consumo de arroz en el país.

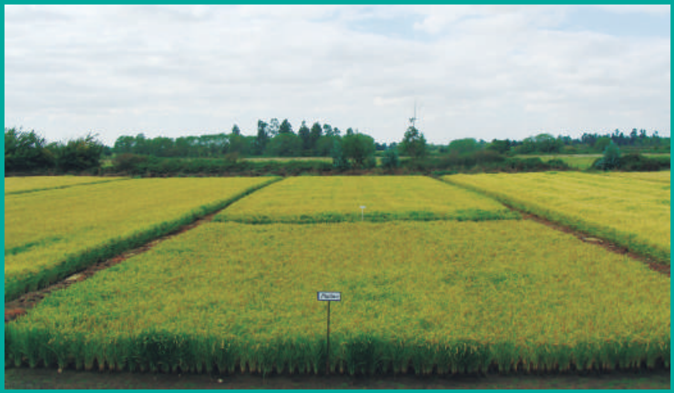

Titular
Instituto de Investigaciones Agropecuarias (INIA).

Registro Solicitado
Chile: SAG - 2015.

Contacto
Johanna Millán L.
Jefa Nacional Unidad Gestión de la Innovación
Teléfono: (+ 56 2) 2577 1024
E-mail: johanna.millan@inia.cl

INIA
Instituto de Investigaciones Agropecuarias
