# Supplementary material for: Protoplast-Based Regeneration Enables CRISPR/Cas9 Application in Two Temperate Japonica Rice Cultivars
Source: Plants (Basel). 2025 Jul 5;14(13):2059. doi: 10.3390/plants14132059 (PMC12251794; doi:10.3390/plants14132059)
Supplement: Supplementary file 1 [file plants-14-02059-s001.zip › 22. Onix-INIA.pdf]

Onix: primera variedad chilena de arroz negro con adaptabilidad al cambio climático

CEREALES

Nuevas variedades vegetales

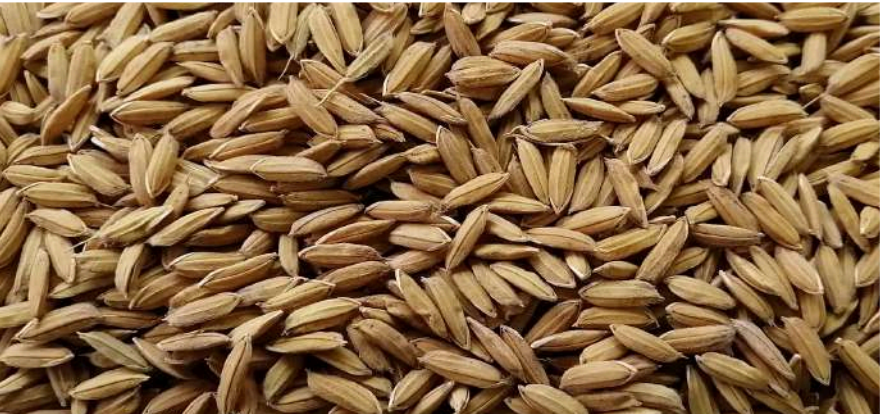

Tecnologías INIA | Unidad Gestión de la Innovación | Instituto de Investigaciones Agropecuarias (INIA) | 2024

INIA
Instituto de Investigaciones Agropecuarias

Onix: primera variedad chilena de arroz negro y adaptabilidad al cambio climático

CEREALES

DESCRIPCIÓN

La nueva variedad de arroz Onix destaca por el color de su grano púrpura, debido a la alta concentración de antocianina; es de tamaño medio y ofrece un rendimiento estable de 71,6 qq/ha. Onix tiene un ciclo fenológico tardío (116,2 días) y combina resistencia al acame, con buena aptitud para cosecha mecanizada, aunque es susceptible al frío. La planta es de altura promedio de 96,9 cm, crecimiento erecto y vigorosa estructura. Su hoja bandera, de color verde claro, se dispone horizontalmente a la panícula, que es de tipo intermedia.

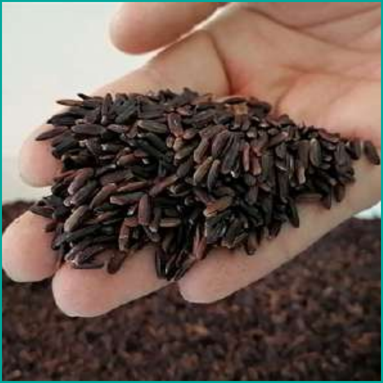

USOS Y APLICACIONES

La variedad Onix es apta para siembra en octubre en toda la zona arrocera nacional, desde Maule hasta Ñuble, y puede cultivarse tanto en siembra directa en seco como en sistemas convencionales con inundación. Su versatilidad favorece a los productores, maximizando la producción en distintas condiciones.

POTENCIAL DE MERCADO

Onix, el primer arroz negro chileno, ofrece a los productores una opción innovadora para diversificar su producción y desarrollar subproductos de alto valor agregado. Su inclusión en rotaciones agrícolas optimiza el uso del suelo, mientras que su alto contenido de fibra y antioxidantes lo posiciona como una alternativa saludable, con gran potencial en mercados especializados y de exportación.

Obtentor
Karla Cordero Lara

Propietario
Instituto de Investigaciones Agropecuarias (INIA)

Representante
Instituto de Investigaciones Agropecuarias (INIA)

Registro Solicitado
Chile SAG - RVP - 2022 - Provisoria

Contacto
Johanna Millán León
Jefa Nacional
Unidad Gestión de la Innovación
E-mail: johanna.millan@inia.cl

INIA
Instituto de Investigaciones Agropecuarias
