# Supplementary material for: Protoplast-Based Regeneration Enables CRISPR/Cas9 Application in Two Temperate Japonica Rice Cultivars
Source: Plants (Basel). 2025 Jul 5;14(13):2059. doi: 10.3390/plants14132059 (PMC12251794; doi:10.3390/plants14132059)
Supplement: Supplementary file 1 [file plants-14-02059-s001.zip › Supplementary Table S1.pdf]

Supplementary Table S1: Preparation of solutions used for *Oryza sativa* L. protoplast isolation and regeneration

Enzyme solution

To prepare the enzyme solution 0.075 g of Onozuka R-10 cellulase, 0.0375 g of Macerozyme R-10, 20 mM MES (pH 5.7), 10 mM KCl, 10 mM of $CaCl_2$ and 0.005 g of BSA were added to 50 mL beaker. The solution was sterilized using a 0.2-μm syringe filter.

FW solution (wash solution)

To prepare 50 mL the FW solution, 0.6 M of mannitol, 2 mM $CaCl_2$, 5 mM of MES (pH 5.7), 0.05 g of BSA and 19.5 mL of H_2O distilled autoclaved. The solution was sterilized using a 0.2-μm syringe filter and stored at room temperature.

1% Sodium alginate solution

To prepare sodium alginate solution, 10 g/L sodium alginate, 7.28 g D-mannitol, 400 µL of $CaCl_2$ 500 mM and 500 µl of MES (pH 5.7) were dissolved in 100 mL of H_2O distilled autoclaved (pH 5.7). The solution was first sterilized in an autoclave and stored at room temperature.

$CaCl_2$ solution (50 mM)

To prepare $CaCl_2$ solution, 54.6 g D-mannitol, 5.5 g $CaCl_2$, 5 mM MES (pH 5.7), 0.5 g BSA and 40 mM of glycine were dissolved in 500 mL of H_2O distilled autoclaved. The solution was sterilized in an autoclave and stored at room temperature.

40% w/v PEG 4000

To prepare 5 mL of PEG 4000 solution, 2 g of PEG 4000 are added to 0.6 M of mannitol, 2 mM of Tris HCl pH 7.5, 5 mM of $CaCl_2$ and 1 mL of H_2O distilled autoclaved. The solution was sterilized using a 0.2-μm syringe filter.

W5 solution

To prepare 20 mL of W5 solution, 2 mM of MES (pH 5.7), 154 mM of NaCl, 5 mM of KCl, 125 mM of $CaCl_2$ and 12.3 mL of H_2O distilled autoclaved. The solution was sterilized using a 0.2-μm syringe filter.

MMG solution

To prepare 5 mL of MMG solution, 4 mM of MES (pH 5.7), 0.4 M of mannitol, 15 mM of $MgCl_2$ and 1.48 mL of H_2O distilled autoclaved. The solution was sterilized using a 0.2-μm syringe filter.
