# Supplementary figures and images for: Protoplast-Based Regeneration Enables CRISPR/Cas9 Application in Two Temperate Japonica Rice Cultivars
Source: Plants (Basel). 2025 Jul 5;14(13):2059. doi: 10.3390/plants14132059 (PMC12251794; doi:10.3390/plants14132059)

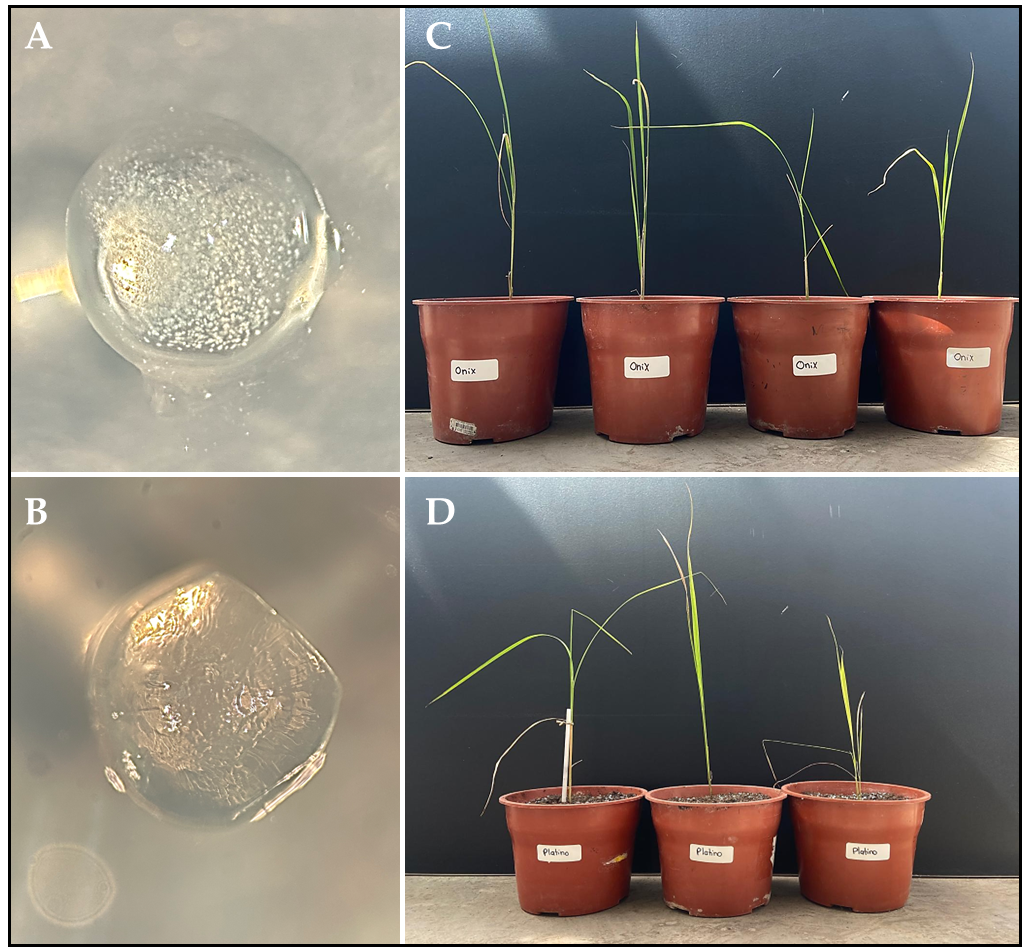

Supplement: Supplementary file 1 [file plants-14-02059-s001.zip › Supplementary Figure S1.png]
